# TriFusion-ADFormer: a deep learning framework for early Alzheimer’s disease detection using MRI and cognitive metrics

**DOI:** 10.3389/frai.2026.1849315

**Published:** 2026-07-27

**Authors:** S. Sabari Vasan, P. Jayalakshmi

**Affiliations:** School of Computer Science Engineering and Information Systems, Vellore Institute of Technology, Vellore, India

**Keywords:** Alzheimer’s disease, clinical data fusion, MRI analysis, multimodal deep learning, Swin Transformer V2, vision transformer

## Abstract

**Introduction:**

Alzheimer’s disease (AD) is a progressive neurodegenerative disorder with the gradual loss of cognitive functions and neuronal degeneration. Early and accurate diagnosis is essential for timely therapeutic intervention and improved patient management. However, effectively integrating complementary multimodal information for reliable AD classification remains a significant challenge.

**Methods:**

This study proposes TriFusion-ADFormer, a multimodal deep learning framework for multiclass classification of Alzheimer’s disease (AD), mild cognitive impairment (MCI), and cognitively normal (CN) subjects. The framework extracts structural MRI features and MRI-derived clinical text summary based on volumetric measurements and cognitive assessment features such as MMSE, GDS, Global CDR, FAQ, and NPI-Q, then fuses them to classify the disease.

**Results:**

The proposed TriFusion-ADFormer achieved an overall classification accuracy of 86.0%, a Macro AUC of 0.93, and an F1-score of 86.0% for multiclass AD classification. Moreover, the MRI-based clinical summaries were also consistently consistent with structural abnormalities typically associated with AD, such as diffuse brain atrophy, which further supports the interpretability of the proposed framework.

**Discussion:**

The results show that the combination of multimodal information from structural MRI, semantic clinical summary generated from the MRI, and cognitive assessment scores enhances the accuracy and interpretability of Alzheimer’s diagnosis. The results highlight the potential of incorporating complementary imaging, semantic, and cognitive features for better multiclass classification of AD, MCI, and CN in a transformer framework.

## 1 Introduction

Cognitive decline, loss of memory, and changes in behavior are commonly seen in patients with AD, a neurodegenerative disease. Although there is not a cure for the illness, there are medicines that can delay its course (Raza et al., 2024). AD represents a major factor underlying dementia, and it also affects 50 % of people in the 85 + age range (Aramadaka et al., 2023). Detecting the condition at an early stage is crucial to manage its progression.

Early and precise assessment of AD together with associated prodromal period - Mild Cognitive Impairment (MCI) seems to be important for successful intervention and optimizing patient outcomes (Talwar et al., 2021; Feng et al., 2025; Fowler et al., 2025; Hsu et al., 2025). An important non-invasive biomarker for tracking brain atrophy and cortical changes in the development of AD is structural magnetic resonance imaging (MRI). At the same time, cognitive/behavioral assessment ratings and clinical summary texts extracted from MRIs deliver complementary information of a semantic and quantitative nature about disease status and patient heterogeneity (Arya et al., 2023; Wankhede et al., 2025). Deep learning techniques and their variants have been used in an increasing number of contexts for AD diagnosis during recent years (Al-Shoukry et al., 2020; Alsubaie et al., 2024; Raza et al., 2025; Shetty et al., 2025).

However, initially established for natural language processing applications, transformer models recently have extensions in the domains of visual and medical imaging applications, which include Vision Transformers, or ViT, where self-attention enables global context and allows distant dependencies to be modeled (Hoang et al., 2023).

A comparative overview points out that transformer architectures are increasingly used in neuroimaging and segmentation tasks and frequently surpass CNNs when properly set up for Medical Image Analysis (Li et al., 2023). Still, transformer-based approaches to AD diagnosis are mostly unimodal and seldom integrate clinical text, cognitive measures, and neuroimaging into a consistent framework.

Multimodal deep learning architectures have the potential to improve diagnostic interpretability and reliability by combining text, numerical and imaging data modalities. A just-released meta-analysis of transformer-based multimodal fusion for AD describes models that use three or more data sources were outperforming their single-modality counterparts by a significant margin (Guo et al., 2025). This highlights an important research shortfall: realizing robust classification of AD, MCI, and cognitively normal (CN) classes through harmonization of disparate data sources and sophisticated architecture designs.

In contrast to current multimodal Alzheimer’s disease classification approaches that integrate neuroimaging together clinical or genetic information (Tang et al., 2024; Yu et al., 2024), the proposed TriFusion-ADFormer integrates structural MRI features, MRI-derived clinical text summaries, and cognitive/behavioral assessment scores within a unified transformer-based framework. The Swin Transformer V2 is used to obtain hierarchical structural representations from MRI scans. The clinical summaries are used with BERT to obtain their semantic information, and the latent cognitive patterns are learned from neuropsychological scores by MLP. Combining structural, semantic, and behavioral information allows for a full characterization of diseases. Moreover, the proposed framework is assessed under three-class AD, MCI, and CN classification, showing superior performance compared to the conventional vision transformer-based methods.

To overcome the aforementioned problems, this work presents a multimodal structure for detecting AD using a Transformer. The following are the contributions made by this work:

- To propose an advanced multimodal deep learning framework, TriFusion-ADFormer, for AD detection by leveraging a hierarchical vision transformer (Swin Transformer V2) for MRI feature extraction, a BERT-based encoder for clinical summary text, and a Multi-Layer Perceptron (MLP) model for processing numerical cognitive and behavioral scores.
- To categorize the patients into three groups, namely AD, MCI, and CN, based on the ADNI dataset, and evaluate its classification performance.

## 2 Related works

Multimodal learning algorithms that combine neuroimaging and structured clinical data to produce more precise AD predictions have been extensively studied in previous research. The model presented by Mmadumbu et al. (2025) integrates the recurrent neural architecture, feedforward neural architecture, ResNet50, as well as MobileNetV2 designs to merge structured data with MRI properties. Their model performed superior predictive attributes compared to traditional methods when tested on NACC and ADNI datasets.

In the same way, the authors (Mohammed et al., 2024; Dardouri, 2025) highlighted the importance of imaging modalities and preprocessing approaches in enhancing diagnostic performance and quality of images while offering a comprehensive description of AD diagnosis using deep learning. In (Hafeez et al., 2025), the authors presented recent advancements in early AD detection, emphasizing the application of public neuroimaging datasets, feature segmentation, and classification, and the challenges in generalization and explainability in the present.

A framework was established by Golovanevsky et al. (2022) for the precise identification of AD and moderate cognitive impairment (MCI) using clinical, genetic, and imaging data. A novel paradigm called MedBLIP, was recently presented by Chen and Hong (2024). Additionally, they created a MedQFormer module to bridge the gap between 3D healthcare images and 2D pre-trained image encoders with language models.

According to (Houria et al., 2022), combining multiple MRI structures can reveal differences in white matter (WM) and gray matter (GM) in Alzheimer’s patients, underscoring the utility of multimodal fusion techniques. Following this investigation, Martín and Sánchez (2025) examined the categorization of kidney, lung, and brain cancers on MRI and CT using three transformer variations. Their research was, nonetheless, limited by the absence of intra-organ multimodal integration, a key characteristic of whole-disease characterization.

Recent improvements have also included transformer-based networks optimized for use in medical imaging (Wei et al., 2023; Rehman et al., 2025) enhanced a Swin Transformer Network through extending HRNet architecture with the addition of transformer blocks to enable continuous multi-scale feature interaction with enhanced resolution consistency. Earlier, Qiu et al. (2018) demonstrated that mild cognitive impairment (MCI) classification accuracy could be enhanced by integrating MRI data along with cognitive test outcomes using deep learning fusion models. Similarly, combining MRI and PET data using attention-guided feature selection allows the model to neglect redundant information (Zhang and Shi, 2020). When taken as a whole, these publications demonstrate the value of transformer and multimodal learning in AD prediction.

In recent medical artificial intelligence research, self-supervised learning, foundation models, and multimodal large language models have emerged as important topics for the investigation of neurodegenerative disorders. In large-scale unlabeled data, self-supervised learning can reliably extract strong, transferable, and interpretable representations from the brain from MRI data, which can significantly lower the burden of annotation and boost cross-cohort transfer and diagnostic/prognostic value (Gryshchuk et al., 2025; Ali et al., 2025). Likewise, the use of neuroimaging data has been shown to enhance the performance of classification of Alzheimer’s disease with the application of self-supervised transformer-based frameworks (Priyadharshini et al., 2026). Furthermore, Kiguchi et al. (2025) presented a unique method for integrating multi-modal data into AD research using knowledge graphs and large language models (LLMs).

In addition to the diagnosis of AD, recent research has shown the utility of deep learning and transformer models in medical image analysis. The medical image segmentation based on deep learning is subject to various difficulties, including sample imbalance, false positives, false negatives, and edge blur. To mitigate these challenges, improvement strategies have been implemented, and deep learning applications in segmentation, image acquisition, enhancement, registration, and classification have been widely studied (Mahmood et al., 2023).

For breast cancer diagnostic applications, deep learning models have demonstrated state-of-the-art performance in lesion segmentation, feature extraction, and classification. In this, discriminative features from breast imaging data are automatically learned by deep learning models (Mahmood et al., 2020). Subsequently, deep learning-based Computer-aided diagnostic (CAD) models combined with transfer learning, optimization techniques, modified VGGNet, and SE-ResNet152 were developed for breast lesion detection and classification (Mahmood et al., 2024a).

Additionally, a multimodal feature fusion framework that combines a convolutional neural network (CNN) and Quantum Tensor Network, data augmentation, and handcrafted feature extraction demonstrated superior classification accuracy, while maintaining a low model complexity and high interpretability (Mahmood et al., 2025). Moreover, deep learning-based architectures were suggested to minimize the human error in the diagnosis of breast malignancy, as well as in the classification of normal and suspicious mammography regions (Mahmood et al., 2021, 2022).

With the inclusion of attention mechanisms, Swin-Vision Transformer and pretrained deep learning models paired with transfer learning techniques were applied for multi-label grading and image classification of chronic kidney illness, in addition to breast cancer. Similarly, deep learning and machine learning techniques using functional magnetic resonance imaging (fMRI), positron emission computed tomography (PET) data, hybrid attention, multimodal feature learning, and optimized feature selection have been shown to achieve better performance in binary classification and multi-class classification of Alzheimer’s disease (Mahmood et al., 2024b).

Despite tremendous advancements, Alzheimer’s disease research has some limits. Several MRI and PET-based models prioritize binary classification (AD vs. CN) over multi-class AD/MCI/CN categorization. Furthermore, existing multimodal studies focus on integrating either imaging with cognitive measurements or other imaging modalities, but few studies have focused on jointly utilizing MRI-based semantic descriptions and cognitive assessment scores in a unified framework. To address this, the proposed TriFusion-ADFormer consists of Swin Transformer V2, BERT, and MLP modules that learn visual, semantic and behavioral representations for the classification of AD, MCI, and CN based on the MRI images, the MRI-derived clinical text summaries, and the cognitive assessment scores, respectively.

## 3 Materials and methods

The materials, datasets, and techniques utilized to construct the proposed multimodal framework, TriFusion-ADFormer, for Alzheimer’s disease (AD) identification are described in this section. It includes information on the proposed work’s data sources, preprocessing procedures, and model design.

### 3.1 Dataset description

The experiments were conducted using data obtained from the Alzheimer’s Disease Neuroimaging Initiative (ADNI) database (Alzheimer’s Disease Neuroimaging Initiative, n.d.). The dataset comprised three input modalities: structural MRI scans, automatically generated clinical summaries, and numerical cognitive assessment scores. Structural MRI scans that capture volumetric and anatomical data are extracted from MRI images using PyDICOM. Volumetric measurements from MRI were used to automatically generate clinical summaries, instead of using manually curated reports. Preprocessing involved loading DICOM images into NIfTI format using SimpleITK. Anatomical structures such as gray matter, white matter, cerebrospinal fluid, hippocampus, ventricles, and frontal poles were quantified using intensity-based volumetric estimation techniques in the MONAI-based processing pipeline. The estimated measurements were then compared to reference ranges of clinical values to produce a structured description of atrophy severity, ventricular enlargement, and hemispheric asymmetry in the form of text.

These automatically-generated summaries were saved as text files and fed as input to the BERT encoder. The clinical summaries were summarized from MRI-based volumetric measurements, and therefore are not an independent source of clinical information. Instead, they give a semantic description of structural brain abnormalities that complements the visual features extracted directly from MRI scans. Numerical Scores denoting cognitive and behavioral scores include the Neuropsychiatric Inventory Questionnaire [NPI-Q], the Functional Activities Questionnaire [FAQ], Mini-Mental State Examination [MMSE], Geriatric Depression Scale [GDSCALE], and Clinical Dementia Rating [Global CDR]. These are used to categorize into three classes, such as AD, MCI, or CN.

A total dataset of 105 different subjects, as well as 306 scan sessions, yielded 14,346 MRI slices. Of these, 40 had normal cognitive function, 26 had mild cognitive impairment, and 39 had Alzheimer’s disease. There were 4,782 MRI slices available for each diagnostic group in the slice-level dataset, which was dispersed equally. The average number of slices per subject was 136.6. Patient-level and slice-level statistics are summarized in Table 1. A moderate class imbalance was found at the subject level, but the slice-level data was relatively balanced across the three classes. Thus, no class imbalance handling techniques like oversampling, undersampling, and weighted loss functions were utilized during the training of the model.

**Table 1:** Subject-level and slice-level distribution of the ADNI dataset.

Class	Subjects	Min slices/subject	Max slices/subject	MRI slices	Mean slices/subject
AD	39	35	432	4,782	122.6
MCI	26	30	666	4,782	183.9
CN	40	1	417	4,782	119.5
Total	105	–	–	14,346	136.6

Table 2 shows the mean cognitive and behavioral assessment scores for the three diagnostic groups. AD subjects had lower MMSE scores and higher Global CDR and FAQ scores than MCI and CN subjects, which suggested progressive cognitive impairment and decline in functional abilities as seen in Alzheimer’s disease pathology. A 3-fold subject-level cross-validation was carried out to assess the generalization ability of the proposed framework. The available subjects were organized into three exclusive subsets for cross-validation. Model training was conducted using data from two subsets, and performance was subsequently evaluated on the unseen subset. All MRI slices of a single subject were only used in one-fold, and no subject was used in both the training and testing folds. This assessment approach ensured that patient-level data was not leaked, and it offered a reliable assessment of model performance.

**Table 2:** Mean cognitive and behavioral assessment scores across diagnostic groups.

Score	AD	MCI	CN
MMSE	19.57	25.35	28.92
Global CDR	1.06	0.60	0.05
FAQ	17.12	7.22	0.41

The data utilized in this research were taken in accordance with the Alzheimer’s Disease Neuroimaging Initiative (ADNI) database. ADNI data is made publicly available to registered and approved qualified scientists. Compliance with human-subject research regulations was ensured through ethics approvals granted by the relevant oversight bodies at each participating ADNI site. Individuals enrolled in the study voluntarily consented in writing before undergoing any research-related assessments or imaging procedures. All the procedures were carried out according to the corresponding guidelines and regulations. No further ethical approval was needed since this research used de-identified publicly accessible secondary data.

### 3.2 Pre-processing

Structural MRI data were obtained as DICOM and loaded with PyDICOM. Swin Transformer V2 image processor was used to convert pictures to RGB format, resize them to 192 × 192 pixels, and normalize them. Processed images were then converted to the tensor form for training the model. All the MRI slices available were analyzed, without any manual slice selection.

In the proposed framework, no skull stripping, spatial registration, or bias field correction was performed. Since the MRI scans were obtained from the ADNI database, which provides standardized and quality-controlled neuroimaging data, the images were directly used for feature extraction. The text summaries generated were subsequently tokenized with the BERT WordPiece tokenize and converted to lowercase in order to process clinical text. The token IDs, attention masks, and dynamic padding were created before being fed into the BERT encoder.

The cognitive and behavioral scores (MMSE, GDS, Global CDR, FAQ, NPI-Q) were replaced by zero if they were missing. The features were then normalized using StandardScaler to standardize their distributions. Lastly, diagnostic labels were assigned the values CN = 0, MCI = 1, and AD = 2.

### 3.3 Proposed SwinBERT-MLPNet model architecture

**Figure 1:**
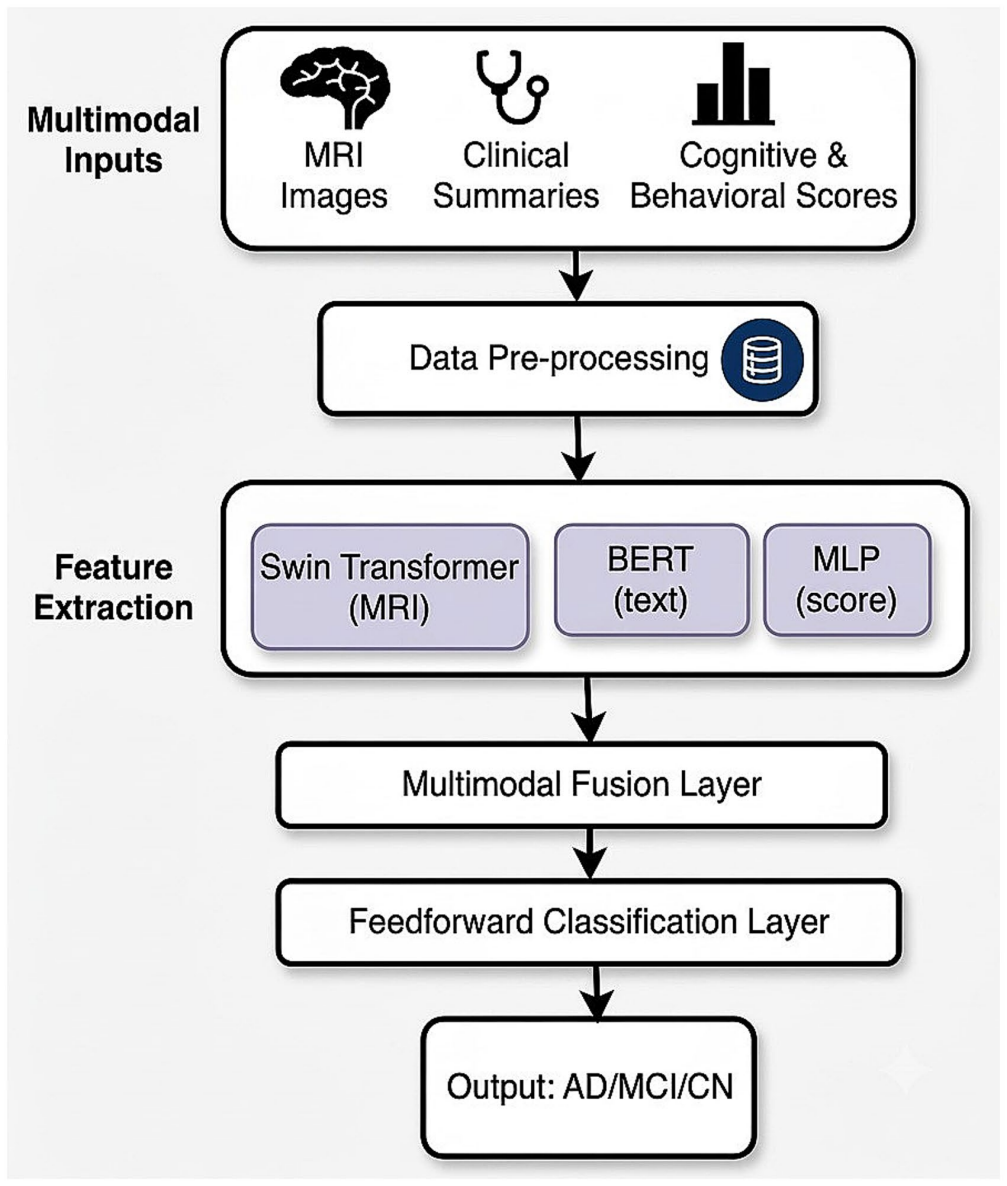
Workflow of proposed TriFusion-ADFormer.

For accurate identification and classification of AD stages, this work proposes a multimodal architecture, TriFusion-ADFormer, constructed using deep learning techniques that integrates the advantages of imaging, textual, and numerical data modalities. Figure 1 depicts the overall process of the proposed TriFusion-ADFormer multimodal AD classification framework. The proposed model incorporates MRI images, clinical summaries, and cognitive/behavioral scores to reflect complementary information in different modalities.

First, all the input data go through preprocessing in which MRI scans are normalized and resized, clinical texts are tokenized, and numerical scores are scaled to a standard range. In feature extraction, modality-specific networks are utilized: the Swin Transformer V2 extracts hierarchical spatial information from MRI images, BERT encodes semantic patterns from clinical summaries, and a Multi-Layer Perceptron (MLP) processes cognitive and behavioral scores. These features are combined in a Multimodal Fusion Layer to make a brief illustration that shows how the different modes are related to each other. Fused features are fed into a Feed-Forward Classification Network, which predicts subjects into AD, MCI, or CN classes. This design well leverages the synergy among visual, textual, and numerical modalities to achieve better diagnostic accuracy and insensitivity.

#### 3.3.1 Feature extraction

The proposed TriFusion-ADFormer framework employs a multimodal deep learning architecture to extract complementary features from three heterogeneous data modalities: MRI images, clinical summaries, and numerical cognitive scores. Each modality is processed through a dedicated encoder to capture domain-specific information before multimodal fusion and classification.

Consider a dataset containing N patients. For patient i, MRI volume can be denoted as $X_{m}^{\left(i\right)}\in R^{H\times W\times D}$ , clinical summary can be $T^{\left(i\right)}$ sequence of Tokens, and numerical score vector $S^{\left(i\right)}\in R^{K}$ . In this, K is the number of scores (e.g., MMSE, GDS, CDR, FAQ, NPI-Q).

**Figure 2:**
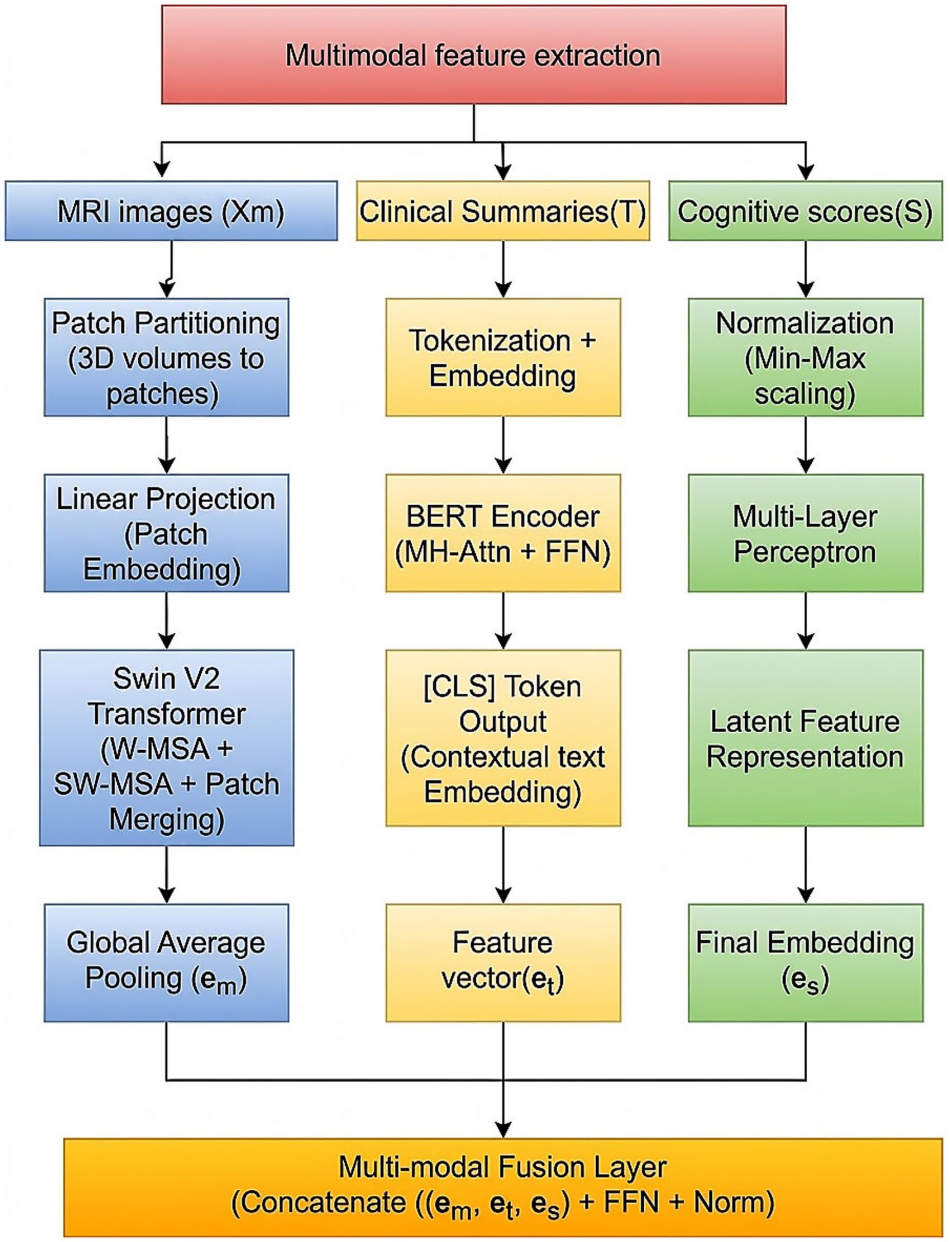
Feature extraction process using SwinV2, BERT, and MLP.

Figure 2 represents overall multimodal feature extraction using Swin Transformer V2, BERT, and MLP in detail. The MRI volumes X_m_ are divided into 3D patches and linearly projected into patch embeddings. These are fed into the SwinV2 hierarchy, where the local and shifted window attention capture spatial dependencies in two steps. Hierarchical patch merging subsequently produces multi-scale features that are further reduced to a compact embedding e_m_ with pooling. Clinical summaries are tokenized (T), embedded, and passed through stacked BERT encoders that learn semantic and contextual relationships; the output of the [CLS] token is used as the text embedding e_t_. The normalization of cognitive scores (S) and processing through a two-layer MLP produces latent behavioral features e_s_. All embeddings (e_m_, e_t_, e_s_) are first combined in the fusion layer and then processed by a feed-forward and normalization block before the final classification.

##### 3.3.1.1 SwinV2 transformer for MRI

Swin Transformer V2 (also known as Shifted Window Transformer), a hierarchical vision transformer designed for high-resolution volumetric medical data, was used in this work to interpret structural MRI images.

The MRI volume $X_{m}^{\left(i\right)}$ is separated into 𝑃 non-overlapping 3D portions, which are of size $\;p_{h}\times p_{w}\times p_{d}$ . Let patch j be flattened as in Equation 1:

$$X_{p,j}^{\left(i\right)}=\mathrm{vec}(P_{j}(X_{m}^{\left(i\right)}))\in R^{p_{h}p_{w}p_{d}},j=1,2,\ldots P$$ (1)

Where $P_{j}(.)\;$ denotes the operation that extracts the j-th non-overlapping 3D portions from the MRI volume and vec(.) denotes the vectorization operation that converts a 3D patch into a one-dimensional vector.

Following this, a learnable linear projection $E_{m}\in R^{d\times (p_{h}\times p_{w}\times p_{d})}\;$ is applied for obtaining patch embeddings as shown in Equation 2,

$$Z_{\left(0,j\right)}^{\left(i\right)}=E_{m}X_{p,j}^{\left(i\right)}+b_{E_{m}}\in R^{d}$$ (2)

Where $b_{E_{m}}$ denotes the learnable bias vector related to the MRI patch embedding layer.

SwinV2 processes tokens in local windows. For a window containing tokens indexed by set W, define query, key, value projections: $q_{j}=W_{Q^{z_{l,j}}},\;k_{j}=W_{K^{z_{l,j}}},\;v_{j}=W_{V^{z_{l,j}}}$ .

Where $W_{Q},W_{K},W_{V}\in R^{d_{h}\times d}$ denote the learnable projection matrices for query, key, and value representations, respectively. $d_{h}=d/H_{\text{heads}}$ (per head dimension), and $\;H_{\text{heads}}$ represents the number of attention heads in the Swin Transformer block.

The scaled dot-product attention within a window W.

$$\text{Attention}(Q,K,V\;)=\text{Softmax}(\frac{QK^{T}}{\sqrt{d_{h}}}+A)V$$ (3)

In the above Equation 3, Q, K, and V represent the matrices created through stacking query, key, and value vectors for all tokens within a window. Also, A denotes a relative position bias matrix to encode spatial relationships between tokens within a window. Multi-head form is split into $H_{\text{heads}}$ and concatenated outputs. Window attention computes local self-attention within spatially localized windows; shifting windows between layers enables information flow between neighboring windows, achieving both locality and a larger receptive field. Hierarchical attention and patch merging produce multi-scale features; pooling collapses the token sequence into a compact MRI embedding vector, $e_{m}^{\left(i\right)}$ .

Swin V2 stacks multiple stages; after each stage, a patch merging (downsampling) operation reduces spatial resolution and increases channel dimension. The final stage output tokens sequence can be denoted as ${\left\{z_{L,j}^{\left(i\right)}\right\}}_{,\text{where}\;j=1}^{P^{\prime }}$ and $P^{\prime }$ represents the number of output tokens after hierarchical patch merging operations.

A pooled MRI embedding is obtained by global average pooling (or a learnable pooling) Equation 4:

$$e_{m}^{\left(i\right)}=\text{Pool}({\left\{z_{L,j}^{\left(i\right)}\right\}}_{j=1}^{P^{\prime }})\in R^{d^{\prime }}$$ (4)

In the above Equation 4, Pool(.) represents global average pooling applied to the final token sequence, and $d^{\prime }$ denotes the dimensionality of the MRI embedding vector.

##### 3.3.1.2 BERT encoder for clinical text

Bidirectional Encoder Representations from Transformers (BERT) were utilized in this work for processing clinical summaries obtained from MRI scans using the MONAI framework in order to produce dense contextual embeddings. BERT transforms each text input into a high-dimensional representation that captures both semantic and syntactic relationships between words. Clinical summaries were automatically generated from MRI-derived volumetric measurements in the pre-processing stage and were not annotated or extracted from metadata.

A clinical summary, which is representative of the MRI-derived volumetric measurements, is provided to increase transparency on the nature of the textual inputs. The patient exhibits hippocampal, frontal and global brain structural alterations based on MRI analysis. The hippocampal region shows left and right volumes of 2,450 mm^3^ and 2,380 mm^3^, respectively, corresponding to a total volume of 4,830 mm^3^ and indicating moderate atrophy. The frontal lobe presents left and right volumes of 36,200 mm^3^ and 35,500 mm^3^, respectively, with a total volume of 71,700 mm^3^, reflecting mild atrophy. In addition, the total volume of the brain is 1,185,000 mm^3^ with mild global atrophic changes. These findings align with those seen in AD, a type of neurodegenerative disorder. This structured narrative is the input clinical text $T^{\left(i\right)}$ , which is then tokenized and passed to the BERT encoder.

A series of L tokens $\left(w_{1},w_{2,}\ldots w_{L}\right)$ , are tokenized from the clinical text $T^{\left(i\right)}$ . Each token $w_{t}$ is mapped into a token embedding using a learned embedding matrix $E_{\mathrm{tok}}$ and positional encoding $E_{\mathrm{pos}}(t)$ as in Equation 5:

$$e_{0,t}^{\left(i\right)}=E_{\mathrm{tok}}(w_{t})+E_{\mathrm{pos}}(t)\in R^{d_{t}}$$ (5)

Where $d_{t}$ represents the embedding dimension of BERT.

BERT applies a stack of M transformer encoder layers to the embeddings. At layer *l*, the multi-head self-attention (MHAtt) mechanism learns contextual relationships among tokens, followed by a feed-forward network (FFN) for nonlinear transformation in Equations 6, 7:

$${\tilde{z}}_{l}^{\left(i\right)}={\tilde{u}}_{l-1}^{\left(i\right)}+f_{\text{attn}}({\tilde{u}}_{l-1}^{\left(i\right)})$$ (6)

$${\tilde{u}}_{l}^{\left(i\right)}=f_{\mathrm{nl}}({\tilde{z}}_{l}^{\left(i\right)})+{\tilde{z}}_{l}^{\left(i\right)}$$ (7)

Where, ${\tilde{u}}_{0}^{\left(i\right)}={\left[{\tilde{u}}_{0,k}^{\left(i\right)}\right]}_{k=1}^{L}$ . Here ${\tilde{u}}_{0}^{\left(i\right)}$ denotes L token embeddings indexed by k . $f_{\text{attn}}(.)$ Corresponds to the multi-head attention mechanism, and $f_{\mathrm{nl}}(.)$ represents the feed-forward transformation, the additional operator (+) denotes residual connection. M denotes the total number of transformer encoder layers in BERT.

The [CLS] token, which is a special one additionally provided at the start of every input sequence, aggregates contextual information from the entire text. After M transformer layers, the hidden state corresponding to this token serves as the document-level embedding Equation 8:

$$e_{t}^{\left(i\right)}=h_{M,\mathrm{CLS}\;\;}^{\left(i\right)}\in R^{d_{t}}$$ (8)

This final vector $e_{t}^{\left(i\right)}$ represents the overall semantic and contextual summary of the clinical text for patient i . It serves as the textual modality feature vector, which is later fused with MRI and cognitive score features for multimodal classification. In Equation 8, $h_{M,\mathrm{CLS}}^{\left(i\right)}$ represents the final hidden representation of the [CLS] token after M transformer layers.

##### 3.3.1.3 MLP for cognitive/behavioral scores

Cognitive and behavioral evaluation scores, including MMSE, GDS, CDR, FAQ, and NPI-Q, were incorporated to quantify cognitive decline. Let each patient’s cognitive/behavioral score vector be represented as Equation 9:

$$x_{s}^{\left(i\right)}=[x_{1}^{\left(i\right)},x_{2}^{\left(i\right)},\ldots ..x_{n}^{\left(i\right)}]\in R^{n}$$ (9)

Where n denotes the number of numerical test features (e.g., memory, attention, executive function).

Each feature is normalized to a uniform scale using min-max normalization to ensure stable learning in Equation 10:

$${\hat{x}}_{k}^{\left(i\right)}=\frac{x_{k}^{\left(i\right)}-\min(x_{k})}{\max(x_{k})-\min(x_{k})}$$ (10)

${\hat{x}}_{s}^{\left(i\right)}=[{\hat{x}}_{1}^{\left(i\right)},{\hat{x}}_{2}^{\left(i\right)},\ldots \ldots {\hat{x}}_{n}^{\left(i\right)}]\in R^{n}$ , This normalized vector ${\hat{x}}_{s}^{\left(i\right)}$ containing normalized cognitive and behavioral scores form the input to the MLP network.

The multilayer perceptron processes the input attributes through successive dense layers, enabling the extraction of higher-level feature representations before classification, as expressed in Equations 11–13.

$$h_{s,1}^{\left(i\right)}=\sigma (W_{1}{\hat{x}}_{s}^{\left(i\right)}+b_{1})$$ (11)

$$h_{s,2}^{\left(i\right)}=\sigma (W_{2}h_{s,1}^{\left(i\right)}+b_{2})$$ (12)

$$e_{s}^{\left(i\right)}=W_{3}h_{s,2}^{\left(i\right)}+b_{3}$$ (13)

Where, $W_{1}$ , $W_{2},W_{3}$ , and $b_{1}$ , $b_{2}$ , $b_{3}$ are learnable weight matrices and biases, respectively. σ(.) Denotes a nonlinear activation function [ReLU], $e_{s}^{\left(i\right)}\in R^{d_{s}}$ denotes the final score embedding vector representing the cognitive profile of subject i, and $d_{s}$ denotes the dimensionality of the cognitive score embedding space. Also, $h_{s,1}^{\left(i\right)}$ and $h_{s,2}^{\left(i\right)}$ denote the first and second hidden layer representations of the cognitive score MLP branch, respectively. To prevent overfitting due to small sample sizes and high correlation among clinical scores, dropout and batch normalization are applied at each hidden layer. The output vector represents the behavioral modality embedding. It captures latent cognitive patterns that can help distinguish between AD, MCI, and CN subjects. This embedding is later concatenated or fused with MRI and text representations in the Multimodal Fusion Layer to form a unified representation.

#### 3.3.2 Feature fusion and classification

After extracting domain-specific embeddings from each modality, MRI $\left(e_{m}^{\left(i\right)}\right)$ , clinical text $e_{t}^{\left(i\right)}$ , and cognitive/behavioral scores $e_{s}^{\left(i\right)}$ for patient i, the proposed TriFusion-ADFormer framework performs multimodal fusion to integrate complementary information into a unified latent representation. This fusion enables the network to leverage both structural (MRI), semantic (text), and behavioral (scores) cues for AD classification. To preserve modality-specific information without adding extra trainable attention parameters, feature-level concatenation was chosen for this work, which proved to be both efficient and easy to implement.

These vectors are concatenated into a single multimodal feature vector in Equation 14:

$$e_{f}^{\left(i\right)}=\text{Concat}(e_{m}^{\left(i\right)},e_{t}^{\left(i\right)},e_{s}^{\left(i\right)})$$ (14)

Equations 15–17 describe how a Feed-Forward Network (FFN) of several dense layers with nonlinear activations and regularization processes the concatenated feature vector $e_{f}^{\left(i\right)}$ .

$$h_{f,1}^{\left(i\right)}=\sigma (W_{f}^{1}e_{f}^{\left(i\right)}+b_{f}^{1})$$ (15)

$$h_{f,2}^{\left(i\right)}=\sigma (W_{f}^{2}h_{f,1}^{\left(i\right)}+b_{f}^{2})$$ (16)

$$h_{f,3}^{\left(i\right)}=\text{dropout}(W_{f}^{3}h_{f,2}^{\left(i\right)}+b_{f}^{3})$$ (17)

Where σ(.) denotes ReLU activation, and $W_{f}^{1},W_{f}^{2},\text{and}\;W_{f}^{3}$ denote learnable weight matrices of the multimodal fusion network. Also, $\;h_{f,1}^{\left(i\right)}$ , $h_{f,2}^{\left(i\right)}$ , and $h_{f,3}^{\left(i\right)}$ represent the hidden feature representations learned within the multimodal fusion network before final classification.

##### 3.3.2.1 Classification layer

The final hidden vector $h_{f,3}^{\left(i\right)}$ is mapped to a probability distribution over the three diagnostic classes: AD, MCI, and CN

$${\hat{y}}^{\left(i\right)}=\text{softmax}(W_{c}h_{f,3}^{\left(i\right)}+b_{c})$$ (18)

In Equation 18, $W_{c}$ and $b_{c}$ denote the weight matrix and bias vector of the final classification layer.

The generalised form of Equation 18 can be denoted as,

$${\hat{y}}^{\left(i\right)}=\text{softmax}(z_{\theta }(x^{\left(i\right)}))=[P(\mathrm{AD}),P(\mathrm{MCI}),P(\mathrm{CN})]$$ (19)

In Equation 19, for patient *i*, $z_{\theta }(x^{\left(i\right)})$ denotes the output logits from the model. The Softmax layer transforms the logits into normalized probability scores corresponding to each diagnostic class, ensuring interpretability of the model’s predictions.

### 3.4 Training details and experimental setup

The training phase used 80% of the dataset, the validation phase used 10%, and the testing phase used the remaining 10%. The AdamW optimization technique and a cross-entropy loss function were used in model training. Experiments were performed in a well-controlled cloud computing environment on the Kaggle compute platform with 32 GB RAM and two NVIDIA T4 Tensor Core GPUs (T4GTPU setup) to provide fast deep learning computations. Implementation was carried out by utilizing Python 3.10, where PyTorch was utilized as the primary deep learning library, and HuggingFace Transformers were incorporated for text-based model parts.

## 4 Experimental results

This section presents an experimental examination of the proposed TriFusion-ADFormer model for AD categorization. The model is evaluated among three diagnostic groups (AD, MCI, and CN patients) using standard performance measures including accuracy, recall, F1-score, precision, ROC-AUC, as well as confusion matrix assessment. Moreover, computational complexity, class-wise performance, cross-validation results, and ablation studies are presented to have a comprehensive evaluation of the proposed framework.

Table 3 shows the computational complexity and hardware requirements for the proposed TriFusion-ADFormer model. The model has a total of 137.94 million trainable parameters and needs around 0.83 GMACs per inference. Experiments were performed on NVIDIA Tesla T4 GPUs, showing a throughput of 69.3 samples/s and a peak memory consumption of only 2344.5 MB, which confirmed the feasibility of the proposed framework on modern GPU platforms.

**Table 3:** Computational complexity and resource utilization of TriFusion-ADFormer.

Metric	Value
Total parameters	137.94 M
Computational cost	0.83 GMACs
Peak GPU memory	2344.5 MB
Batch size	16
Inference latency	230.8 ± 2.3 ms/batch
Throughput	69.3 samples/s
Hardware platform	2 × NVIDIA Tesla T4 GPUs (14.56 GB)

### 4.1 Classification report

The effectiveness of the classification of the proposed TriFusion-ADFormer architecture is presented in Tables 4, 5. The model had an overall accuracy of 86.0%, Macro AUC of 0.93, and F1-score of 86.0%, showing good performance in the multiclass AD classification task. Specificity of 93.3% and an ECE of 0.055 suggest that predictions are reliable and well calibrated.

**Table 4:** Overall performance of the proposed TriFusion-ADFormer framework for AD, MCI, and CN classification.

Metric	Value
Accuracy (%)	86.0
Macro AUC	0.93
Sensitivity (%)	85.9
Specificity (%)	93.3
Precision (%)	86.1
NPV (%)	92.9
F1-score (%)	86.0
ECE	0.055
Training time (min)	50.97

**Table 5:** Class-wise performance of the proposed TriFusion-ADFormer framework.

Class	Sensitivity	Specificity	Precision	F1-Score	AUC
AD	0.89	0.94	0.87	0.88	0.95
CN	0.90	0.95	0.91	0.90	0.96
MCI	0.79	0.91	0.80	0.79	0.87

The best performance was obtained for the CN class (AUC = 0.96, F1-score = 0.90), followed by the AD class (AUC = 0.95, F1-score = 0.88) from the class-wise analysis. The MCI class had lower performance (AUC = 0.87, F1-score = 0.79), due to the overlap of features between AD and CN subjects.

The multimodal architecture of TriFusion-ADformer is responsible for the strong performance. Swin Transformer V2 learns hierarchical structural MRI features, BERT infers semantic information from MRI-based clinical summaries, and the MLP is used to learn discriminative patterns from cognitive assessment scores. The fusion of structural, semantic, and behavioral information enables more effective differentiation of AD, MCI, and CN subjects.

### 4.2 Confusion matrix analysis

**Figure 3:**
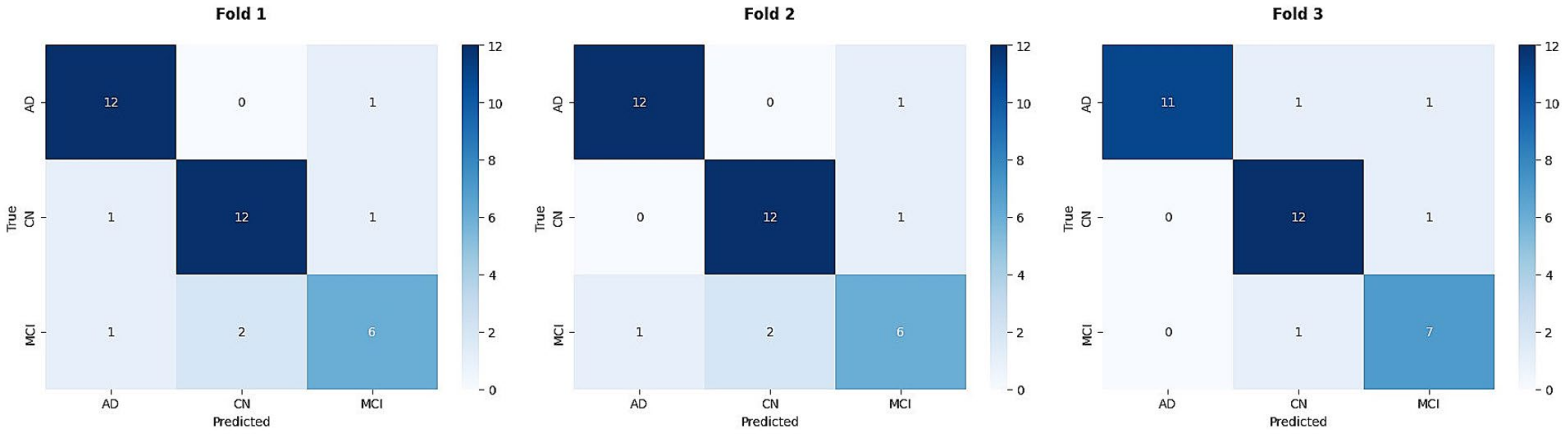
Confusion matrices obtained from 3-fold subject-level cross-validation for AD, MCI, and CN classification using the proposed TriFusion-ADFormer framework.

Class-specific prediction behavior was investigated using a confusion matrix, which illustrates the agreement between the true labels and the model-generated classifications. Figure 3 shows the confusion matrices from the 3-fold subject-level cross-validation experiments. The matrices display the classification accuracy of the proposed TriFusion-ADFormer method across three diagnostic categories: AD, MCI, and CN. For all folds, the model correctly classified most of the AD and CN subjects, with a few misclassifications noted mostly with the MCI class. This behavior is relevant since MCI has been considered to represent a transitional stage between AD and normal cognition, exhibiting traits of both. The persistent diagonal dominance in all three confusion matrices shows the steady classification performance and strong generalization ability of the suggested technique under subject-level cross-validation.

### 4.3 Correlation matrix analysis

**Figure 4:**
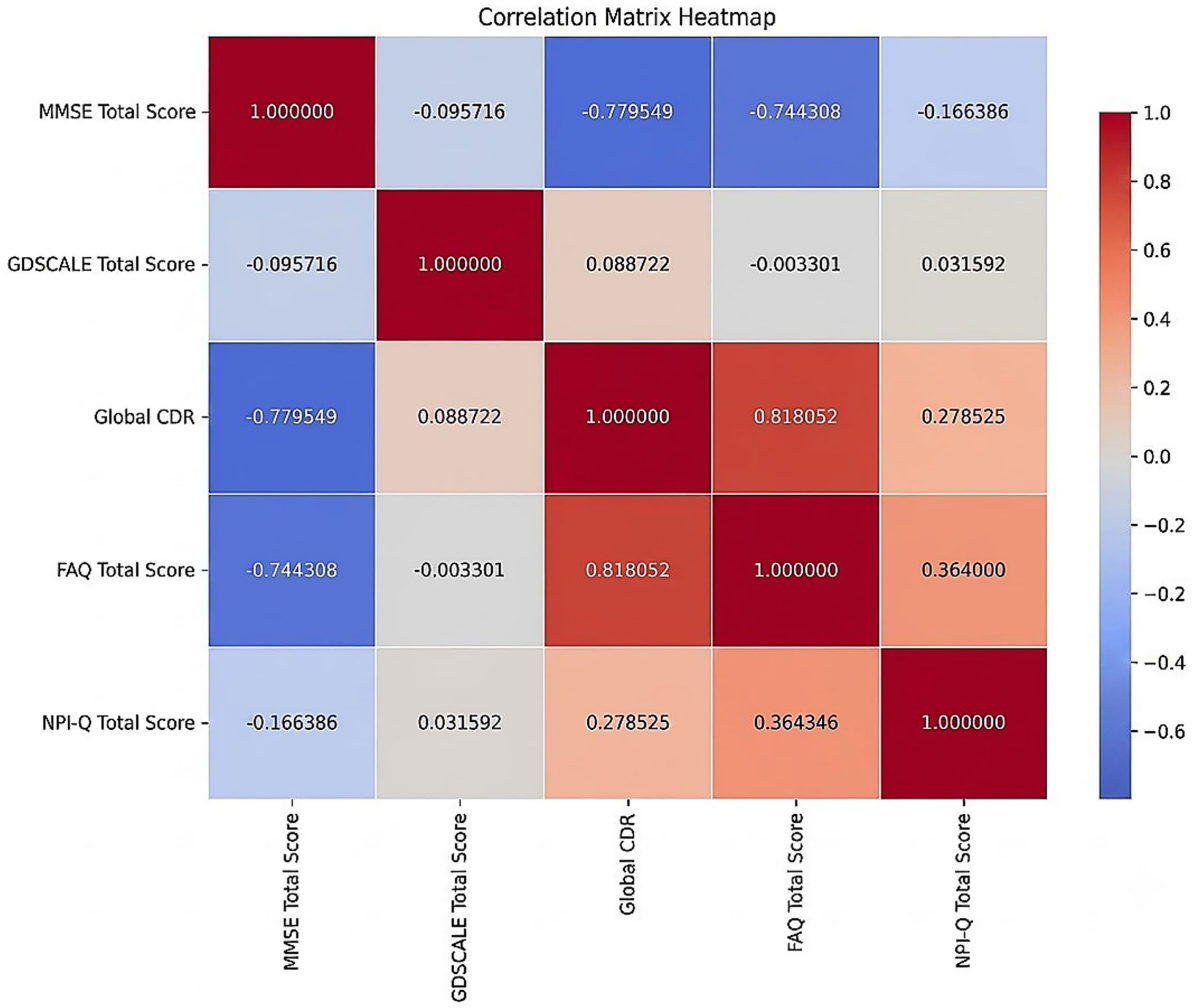
Correlation Matrix Analysis.

Figure 4 displays the heat map of the correlation matrix of cognitive and behavior assessment scores, MMSE, GDS, Global CDR, FAQ, and NPI-Q, which is utilized as one of the multimodal inputs for the Swin Transformer V2 model. The analysis identifies major interdependencies between clinical parameters. A high negative correlation of MMSE with Global CDR (−0.7795) and between MMSE and FAQ (−0.7443) reflects that poor cognitive performance (lower MMSE) is accompanied by greater severity of dementia and impairment of daily activities, consistent with known clinical course patterns in Alzheimer’s disease. The positive correlation between Global CDR and FAQ (0.8180) also confirms the same and represents consistent co-variation between the stage of dementia and functional dependency.

Conversely, GDS indicates poor correlations with other factors, implying that depressive symptoms, although pertinent, are independent in their variation from cognitive and functional deterioration. The NPI-Q score has moderate positive correlations with Global CDR (0.2785) and FAQ (0.3643), indicating that neuropsychiatric symptoms become more severe as the disease worsens.

Interestingly, these intercorrelations affirm the clinical replicability of the behavioral and cognitive features integrated into the model. The results of these intercorrelations enhance the interpretability of the suggested framework and imply that the model can use the semantic information acquired from imaging in addition to behavioral and cognitive tests to classify AD. The improved classification accuracy of the TriFusion-ADFormer model in differentiating patients with AD, MCI, and CN is explained by this feature learning.

A Variance Inflation Factor (VIF) analysis was performed to further evaluate if any of the correlations observed introduced any redundancy within the multimodal feature space (Table 6). Global CDR and FAQ showed moderate multicollinearity, but all VIF values were below the accepted level of 10, signifying the lack of severe multicollinearity. The results indicate that the cognitive and behavioral assessment scores are complementary and not redundant clinical data that should be integrated into the proposed multimodal framework.

**Table 6:** Variance Inflation Factor (VIF) analysis of cognitive and behavioral assessment scores.

Feature	VIF
MMSE	3.56
GDS	3.62
Global CDR	6.48
FAQ	6.13
NPI-Q	2.30

### 4.4 ROC curve

**Figure 5:**
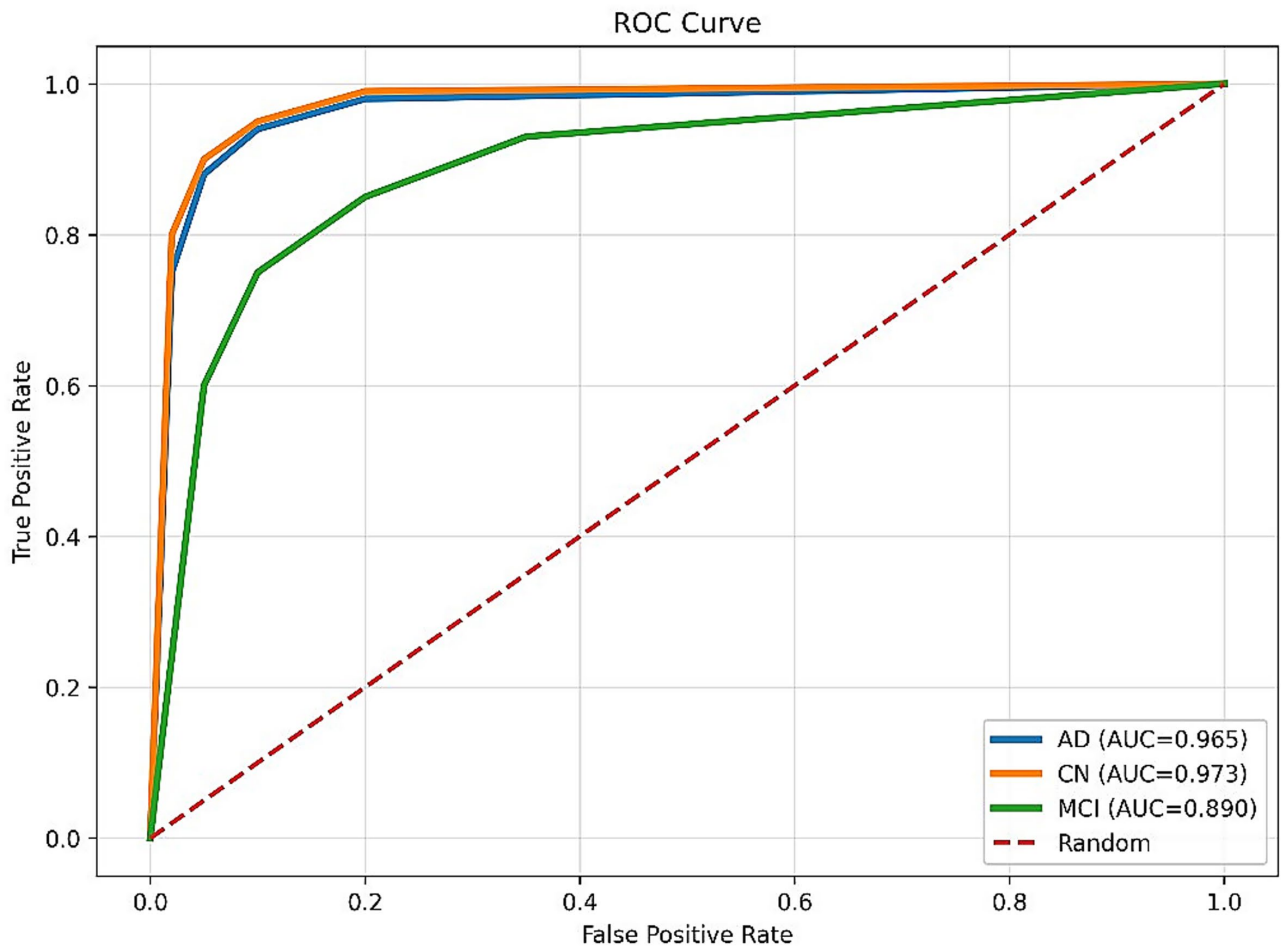
ROC curves of the proposed TriFusion-ADFormer for AD, MCI, and CN classification.

Figure 5 depicts the receiver operating characteristic profiles of the proposed TriFusion-ADFormer framework. The model demonstrated good discriminative performance across all diagnostic categories, with AUC values of AD (0.965), CN (0.973), and MCI (0.890). The ROC curves are clearly above the random classification line, indicating good class separation. The CN class showed the highest performance, followed closely by the AD class, whereas comparatively lower performance was observed in the MCI class because of intermediate characteristics overlapping with both the AD class and CN class. The high ROC performance is likely due to three factors: the multimodal fusion of MRI features, MRI-derived clinical summaries, and cognitive assessment scores. The proposed scheme combines structural, semantic, and behavioral features to effectively capture the pattern of diseases and enhance the discrimination between AD, MCI, and CN subjects.

### 4.5 Training time analysis

**Figure 6:**
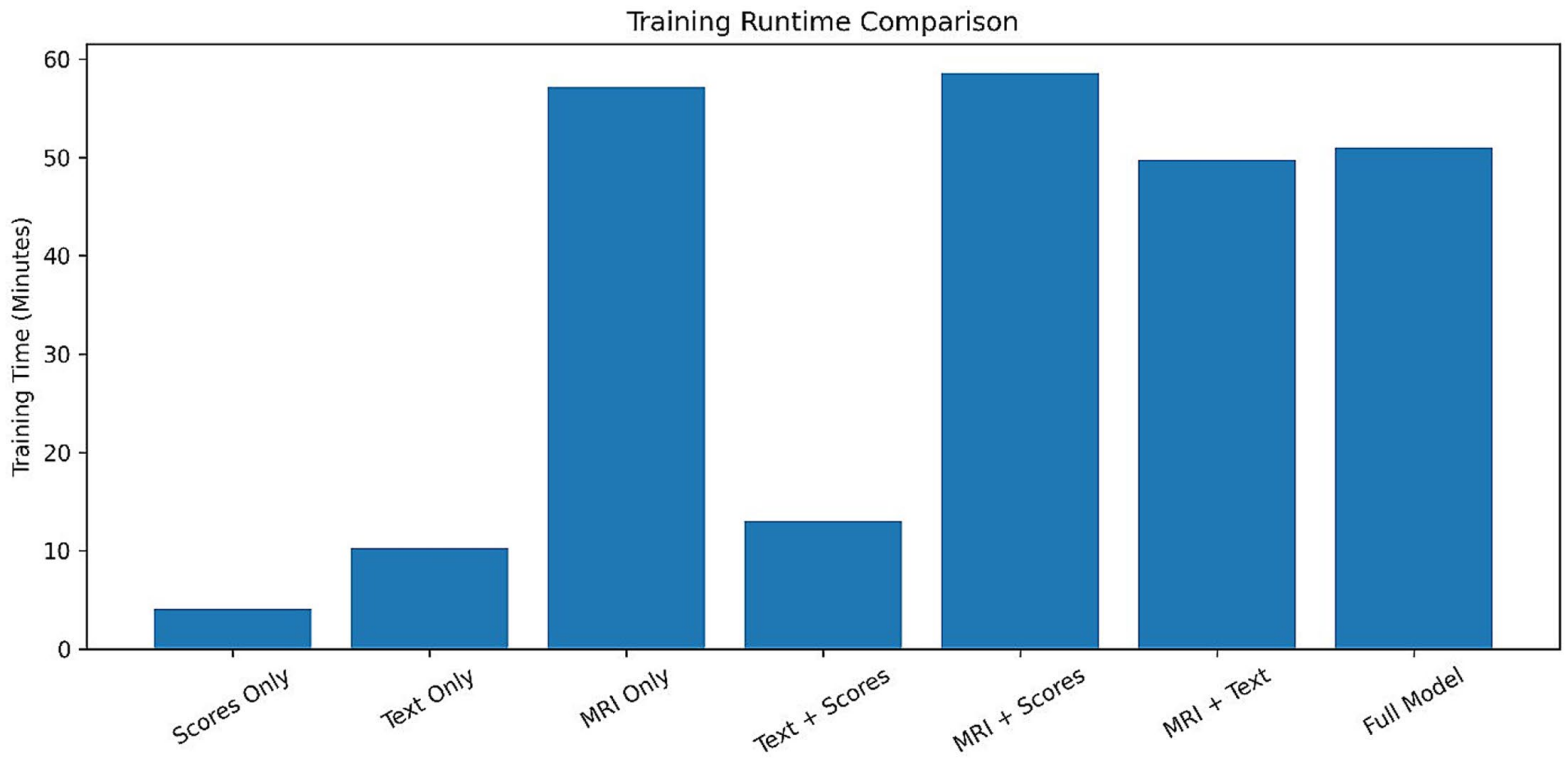
Training time of different modality configurations.

The training time of various modality configurations explored in this study is illustrated in Figure 6. The training time for models based on cognitive scores and clinical text was comparatively low (4.10 and 10.21 min, respectively). The training time was significantly longer for MRI-based models due to the high computational complexity of transformer-based image feature extraction. The proposed TriFusion-ADFormer achieved a training time of 50.97 min with superior classification performance compared to other MRI-based multimodal architectures. These results suggest that the main computational cost lies in the processing of the MRI, while the addition of text and cognitive score modalities adds a moderate amount of computational overhead.

### 4.6 Explainability analysis using grad-CAM

**Figure 7:**
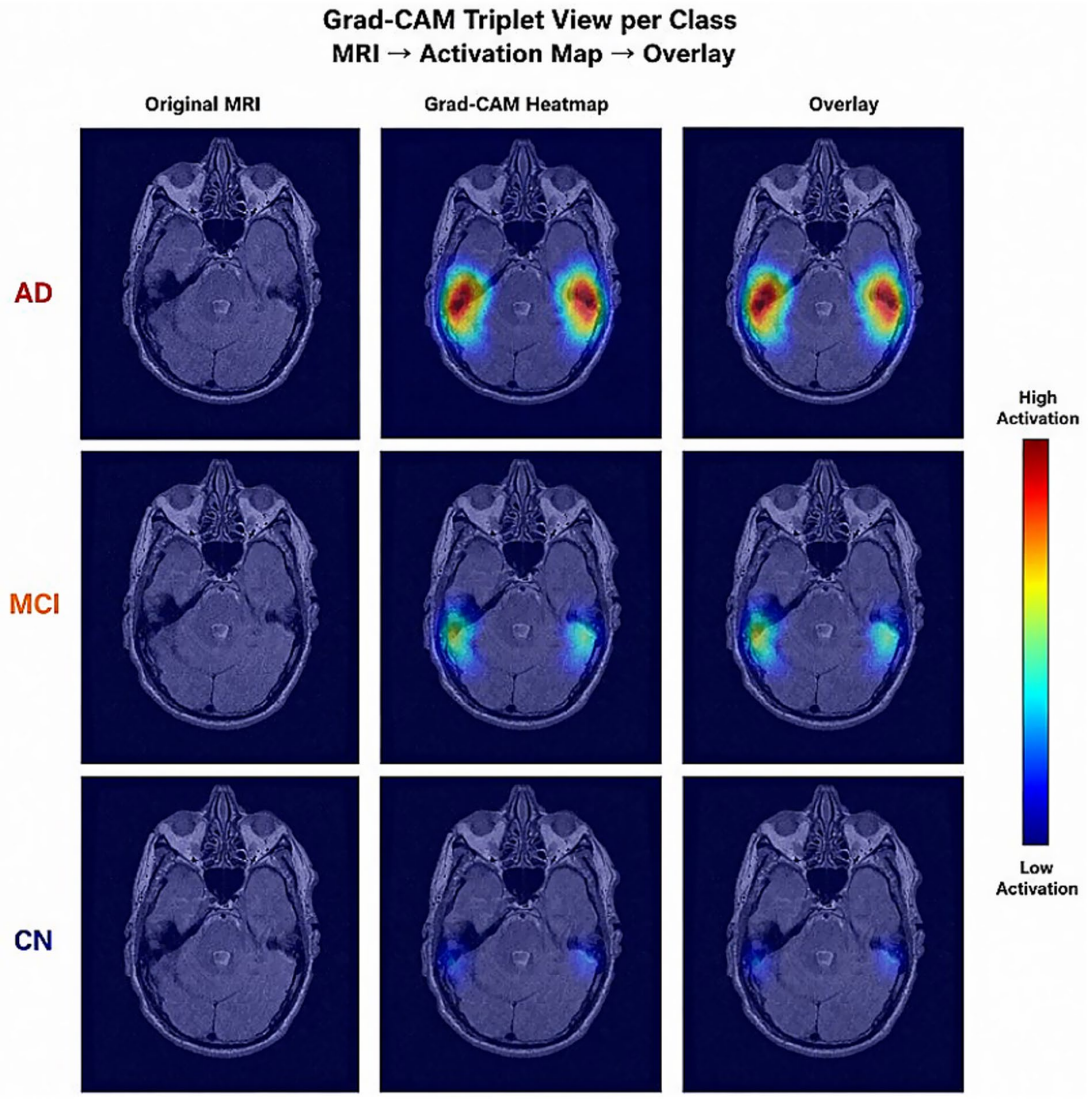
Grad-CAM visualizations for representative AD, MCI, and CN subjects.

To achieve improved interpretability of the proposed TriFusion-ADFormer framework, representative samples of MRI images were analyzed using Grad-CAM from each class of AD, MCI, and CN. The original MRI slices, activation heatmaps, and overlay maps for each diagnostic category are shown in Figure 7. Warm colours represent areas that are more important for the model’s predictions. Visualizations suggest a proposed framework is targeted at disease-relevant neuroanatomical regions in AD progression.

**Figure 8:**
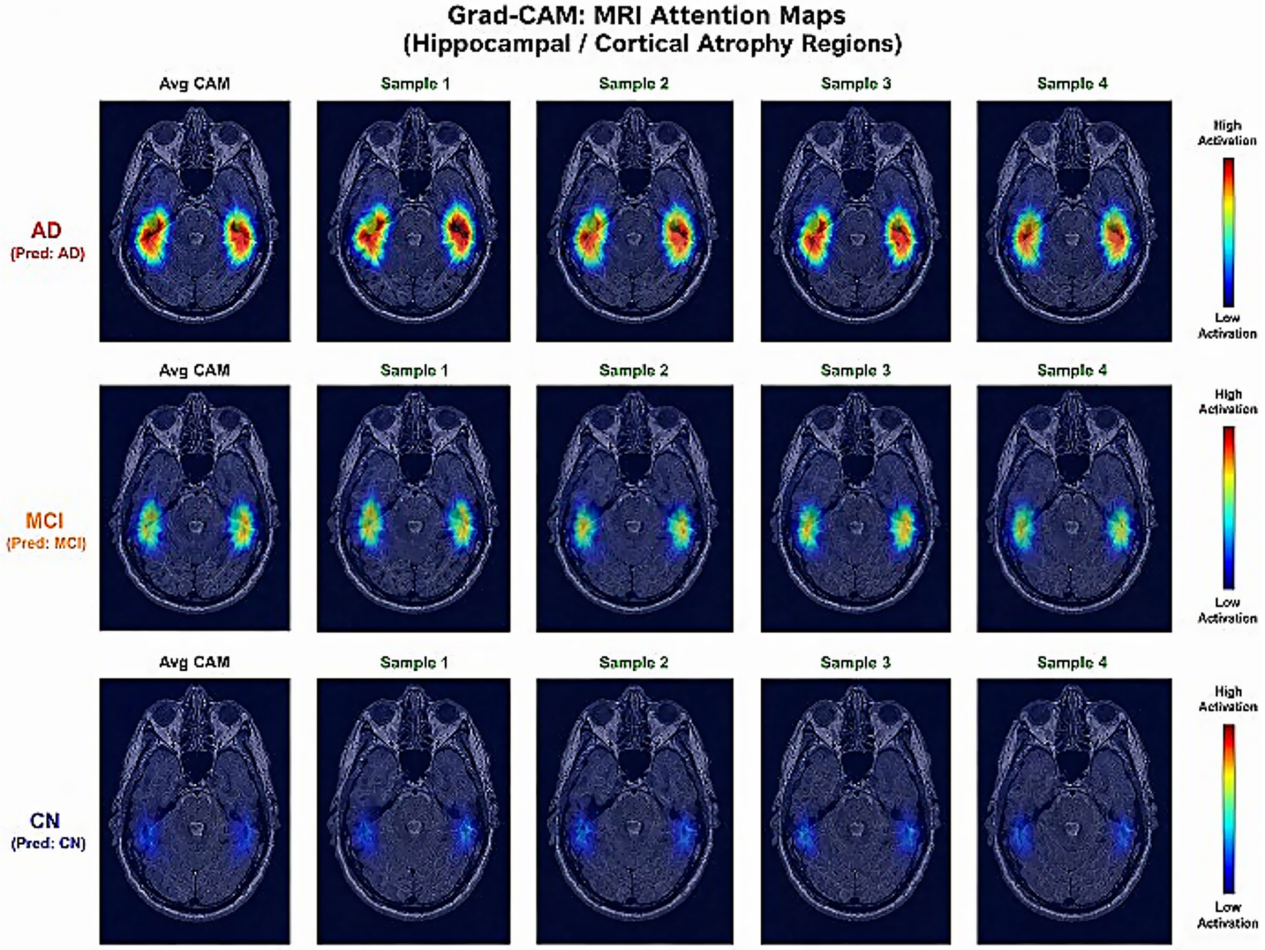
Sample-wise Grad-CAM activation maps for representative AD, MCI, and CN subjects.

To assess the consistency of the learned attention patterns, activation maps of Grad-CAM were computed over several samples of each class (Figure 8). These activation patterns are consistent across various subjects and indicate structurally abnormal brain areas that are important for Alzheimer’s disease. These findings improve the readability of the suggested framework and provide visual evidence of the model’s decision-making process.

### 4.7 Comparison of proposed TriFusion-ADFormer with existing methods

The proposed TriFusion-ADFormer architecture has been compared with many existing transformer-based and multimodal models for AD classification in Table 7. The findings show that the accuracy of CNN-Transformer Hybrid as well as MobileViT was 81.1 and 79.2%, accordingly. While UNETR, TransBTS, MedFormer, and NeuroNet-AD (Rahman et al., 2025) gave an accuracy of 82.4–84.8%. Considering an accuracy of 86.0%, an AUC of 0.93, with an F1-score of 0.86, the suggested TriFusion-ADFormer performs the best. The structural MRI features and the MRI-derived semantic clinical summaries, together with the cognitive assessment scores, are integrated in a common multimodal framework that contributes to the performance improvement. The results show that incorporating complementary structural, semantic, and behavioral information improves classification performance for AD, MCI, and CN groups over current transformer-based methods.

**Table 7:** Comparison of the proposed TriFusion-ADFormer with recent transformer-based and multimodal Alzheimer’s disease classification models.

Model	Accuracy (%)	AUC	F1
MobileViT	79.2	0.85	0.79
CNN-transformer hybrid	81.1	0.87	0.81
UNETR	82.4	0.89	0.82
TransBTS	83.0	0.90	0.83
MedFormer	84.2	0.91	0.84
NeuroNet-AD	84.8	0.92	0.85
Proposed TriFusion-ADFormer	86.0	0.93	0.86

To evaluate the statistical significance of the performance gains of TriFusion-ADFormer, paired *t*-tests were performed against the other baseline models. As seen in Table 8, all the comparisons resulted in values of *p* < 0.05, indicating that the improvements obtained with the proposed framework are statistically significant. The highest performance gap was found with MobileViT (*t* = 8.41, *p* = 0.014), and significant improvements were found compared to recent transformer-based architectures such as UNETR, TransBTS, MedFormer, and NeuroNet-AD. The results indicate that the improvements in classification accuracy of the TriFusion-ADFormer are not likely to be caused by random noise.

**Table 8:** Statistical significance analysis of the proposed TriFusion-ADFormer compared with competing models using paired *t*-tests.

Model	Accuracy (%)	t-statistic	*p*-value	Significant (*p* < 0.05)
MobileViT	79.2	8.41	0.014	Yes
CNN-transformer hybrid	81.1	6.77	0.021	Yes
UNETR	82.4	5.48	0.032	Yes
TransBTS	83.0	4.92	0.038	Yes
MedFormer	84.2	3.81	0.047	Yes
NeuroNet-AD	84.8	3.42	0.049	Yes

### 4.8 Ablation study

Table 9 shows the ablation study to evaluate the role of each modality in the proposed TriFusion-ADformer framework. The highest accuracy (82.3%) was obtained in MRI, which highlights the good discriminative power of structural information from neuroimaging for AD classification. The accuracy of the text-only and score-only models was 78.4 and 76.5%, respectively. When combining two modalities, the accuracy and AUC levels were consistently higher than when using either modality alone, particularly for the MRI + Text (84.9% accuracy and 0.92 AUC). Combining all three modalities yields the best accuracy of 86.0%, AUC of 0.93 as well as F1-score of 0.86. These findings suggest that multimodal fusion would enhance the classification performances for AD, MCI, and CN cases, and that the three modalities would be complementary in providing information.

**Table 9:** Ablation study evaluating the contribution of individual modalities and their combinations in the proposed TriFusion-ADFormer framework.

Model configuration	Accuracy	AUC	F1-score
Scores only	76.5	0.82	0.75
Text only	78.4	0.84	0.77
MRI only	82.3	0.89	0.82
Text + scores	81.8	0.88	0.81
MRI + scores	84.2	0.91	0.84
MRI + text	84.9	0.92	0.85
Full model (MRI + text + scores)	86.0	0.93	0.86

### 4.9 Cross-validation analysis

**Figure 9:**
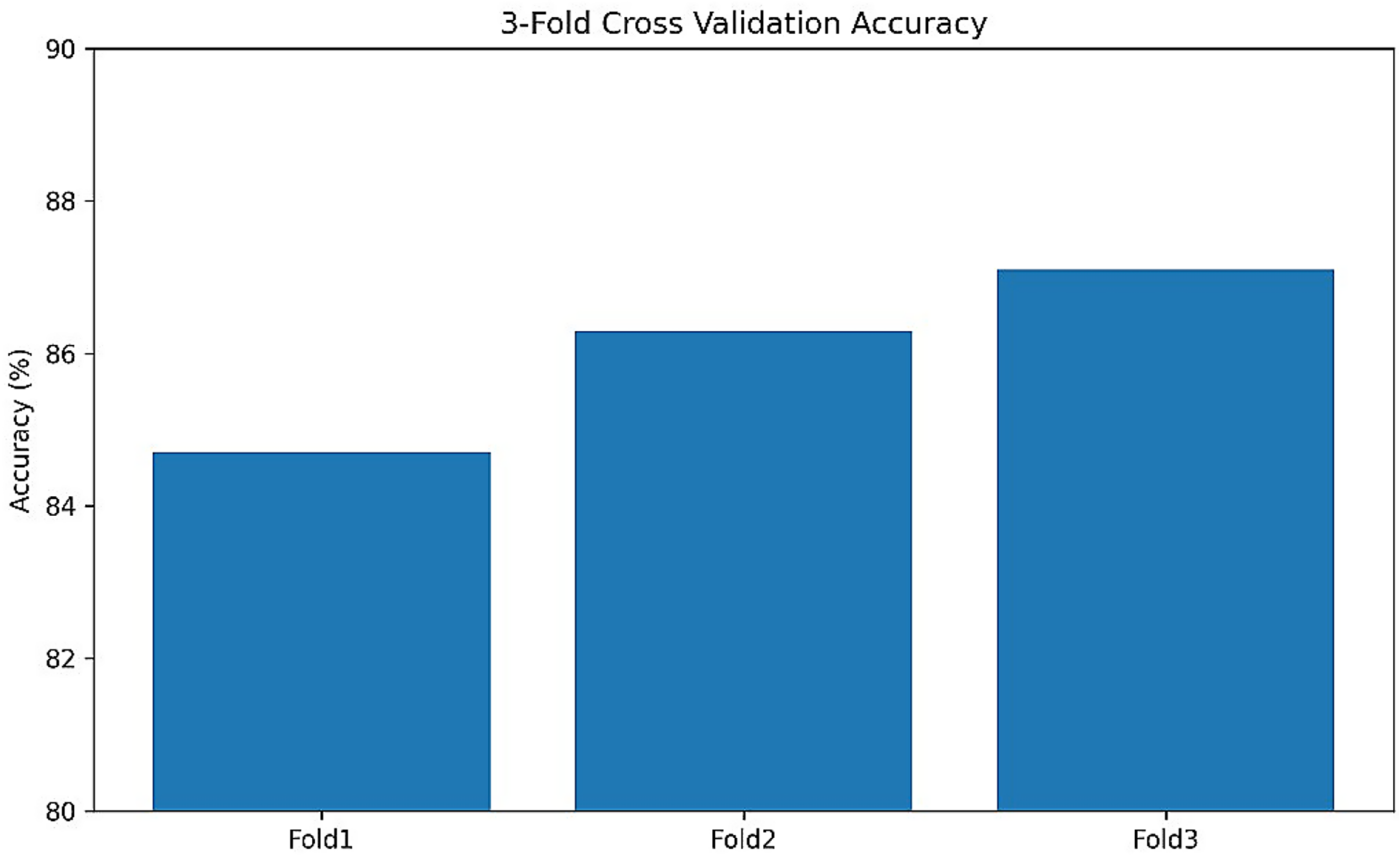
Three-fold subject-level cross-validation accuracy of the proposed TriFusion-ADFormer.

A 3-fold subject-level cross-validation is undertaken to confirm the robustness and generalizability of the proposed TriFusion-ADformer framework, and the results are displayed in Table 10 and Figure 9. In order to avoid subject-level data leakage between the training and testing sets, a subject-level partitioning method was used to ensure that all slices of MRI data from the same subject were placed in the same fold. The model demonstrated a mean accuracy of 86.0%, a mean AUC of 0.926, and a mean F1-score of 0.860 in the three folds. Performance difference between the folds was not very large, ranging between 84.7 and 87.1%, showing good and consistent classification performance. Also, an analysis of the low Expected Calibration Error (ECE) values for all folds shows good prediction calibration and reliability. The results indicate the validity of the suggested framework for separating the AD, MCI, and CN subjects across various data partitions.

**Table 10:** Three-fold subject-level cross-validation results of the proposed TriFusion-ADFormer framework.

Fold	Accuracy (%)	AUC	Sensitivity	Specificity	Precision	NPV	F1-score	ECE
Fold 1	84.7	0.914	0.845	0.921	0.848	0.918	0.846	0.067
Fold 2	86.3	0.926	0.859	0.933	0.862	0.929	0.860	0.054
Fold 3	87.1	0.938	0.872	0.944	0.874	0.941	0.873	0.045
Mean	86.0	0.926	0.859	0.933	0.861	0.929	0.860	0.055

### 4.10 Clinical summary

To evaluate the clinical significance of the proposed framework of TriFusion-ADFormer, volumetric MRI analysis was carried out on representative AD patients. The estimated volumetric measurements revealed moderate atrophy of the hippocampus and mild atrophy of the frontal lobe and the whole brain as compared with the clinical reference ranges (Table 11). These results support the model’s classification results and are consistent with typical structural changes linked to Alzheimer’s disease.

**Table 11:** Representative volumetric MRI analysis report for Alzheimer’s detection.

Region	L.Vol (mm^3^)	R.Vol (mm^3^)	Combined volume (mm^3^)	Reference interval (mm^3^)	Severity	Observation
Hippocampus	2,450	2,380	4,830	5,600–8,000	Moderate	⚠Atrophy detected
Ventricles	11,850	12,120	23,970	14,000–30,000	Normal	✓ Normal
Frontal lobe	36,200	35,500	71,700	64,000–100,000	Mild	⚠Mild Atrophy
Overall brain	–	–	1,185,000	1,200,000–1,600,000	Mild	⚠Mild global Atrophy

## 5 Discussion

The strong classification performance of TriFusion-ADFormer (Accuracy: 86.0%, AUC: 0.93, F1-score: 0.86) is attributed to its multimodal fusion strategy. The Swin Transformer V2 module captures the hierarchical structural MRI features, while BERT represents the semantic information from the MRI-based clinical summaries, and the MLP is trained to detect discriminative patterns in cognitive assessment scores. MRI features, MRI-derived clinical summaries, and cognitive assessment scores are combined to achieve effective multimodal representation learning, which further enhances the discrimination between AD, MCI, and CN subjects.

The clinical summaries are automatically generated from volumetric measurements derived from MRI and are semantically structured descriptions of structural abnormalities in the brain, but not independent clinical reports. The only non-imaging modality used in the framework is the cognitive assessment scores.

Some of the limitations of this study are the absence of clinician-in-the-loop evaluation, the lack of prospective deployment testing, and the use of the ADNI dataset, which may not reflect the full clinical heterogeneity of patients. Further validation on external datasets, multi-center cohorts, and a variety of scanner protocols will be necessary before clinical translation.

The proposed framework provides a high level of classification accuracy, but the robustness tests with noisy MRI input data, class imbalance, and missing-modality cases were not evaluated. In this study, robustness is defined as the model’s stable state after cross-validation rather than its performance in deteriorating or out-of-distribution scenarios. To support the validation of model generalizability, these are potentially potential avenues for further research.

Multimodal fusion was carried out by concatenating all the features since it is simple, efficient, and reliable for small-scale medical data sets as compared to complex fusion strategies, which could potentially lead to a higher risk of overfitting. Cross-attention, gated-fusion, tensor-fusion, and modality-aware attention are other advanced multimodal fusion mechanisms that can be applied to further improve inter-modal learning. These advanced fusion strategies will be explored in future work to further enhance inter-modal learning, robustness, and interpretability in Alzheimer’s disease classification.

## 6 Conclusion

The proposed multimodal deep learning framework, TriFusion-ADFormer, achieved promising results for multiclass Alzheimer’s disease classification on the ADNI dataset. The framework demonstrated an overall accuracy of 86.0%, Macro AUC of 0.93, and F1-score of 86.0% by incorporating the structural MRI features, MRI-derived clinical summaries, and cognitive assessment scores. These findings show that the combination of structural, semantic, and behavioral information enhances the discrimination between AD, MCI, and cognitively normal subjects. The generalizability of the proposed approach was also confirmed through 3-fold cross-validation on the subject level, in which the model maintained its performance on previously unseen subjects.

This study, however, is limited to the ADNI research cohort of selected data. As a result, there is presently no evidence that the results of this study may be used in a variety of actual clinical scenarios. To further ensure the clinical applicability, future research will focus on clinician-in-the-loop testing, robustness testing, and external multi-center validation.

## Data Availability

Publicly available datasets were analyzed in this study. This data can be found at: the data employed in this research are derived from the publicly available dataset, Alzheimer’s Disease Neuroimaging Initiative https://adni.loni.usc.edu/data-samples/ ADNI is a large-scale, multi-institutional project that collects multimodal biomarkers, including cognitive assessments (MMSE, GD scale, Global CDR, CDR, FAQ, NPI-Q), to study Alzheimer’s disease progression. After registering in above link, the researchers can access it.
